# Health Disparities by Type of Disability: Health Examination Results of Adults (18-64 Years) with Disabilities in Shanghai, China

**DOI:** 10.1371/journal.pone.0155700

**Published:** 2016-05-19

**Authors:** Qi Kang, Gang Chen, Jun Lu, Huijiong Yu

**Affiliations:** 1 School of Public Health, Fudan University, Shanghai 200032, P. R. China; 2 China Research Center on Disability Issues at Fudan University, Shanghai 200032, P. R. China; 3 Department of Rehabilitation, Shanghai Disabled Persons’ Federation, Shanghai 200126, P. R. China; University of Perugia, ITALY

## Abstract

**Aims:**

There have been few studies on the disparities within the population with disabilities, especially in China. The aim of this study was to evaluate the differences in some health conditions among people with different types of disabilities in Shanghai.

**Methods:**

This study was conducted using data from the Shanghai Disabled Persons’ Rehabilitation Comprehensive Information Platform. The records of 31,082 persons with disabilities who had undergone professional health examination were analyzed, and the prevalence and number of five diseases and five risk factors were examined. Logistic regression was used to explore disparities from two perspectives: 1) basic differences, unadjusted for other factors, and 2) differences after adjusting for key demographic covariates. A p-value < 0.05 was considered significant.

**Results:**

Individuals with visual disability had a high rate of refractive error (60.0%), and averaged 1.75 diseases of interest, which was the highest value among all disability types. The mean number of risk factors we measured was greatest (1.96) in the population with mental disability. There were significant differences (*p* < 0.05) between the hearing and speech impairment group and the other groups with respect to most health outcomes, except chronic pharyngitis, hepatic cysts, and high blood pressure.

**Conclusion:**

Significant differences of selected health outcomes between groups with different types of disabilities remained after controlling for key demographic indicators. Further research is needed to explore the relationships between health conditions and disability types.

## Introduction

Health disparities refer to clinically and statistically significant differences in health outcomes between individuals or groups of individuals [1]. Public health research has a long history of uncovering health disparities between different population groups. A wide variety of disparity factors have been studied at the individual, social, and systemic levels, to find the target populations that are at disadvantage with respect to a particular health issue. After detecting and understanding the disparities, intervention can be implemented for specific groups. Health disparity research plays a key role in resource allocation, especially for vulnerable populations [2].

People with disabilities are those who have long-term physical, mental, or intellectual impairments that, together with various barriers, may hinder their full and effective participation in the society, at the same level as other people. It is unquestioned that people with disabilities constitute one of the most socially excluded groups in any society [3]. The World Report on Disability, issued in 2011 by the World Health Organization (WHO) and World Bank, reveals that more than one billion people in the world, about 15% of the global population, live with some form of disability [4]. Because of the large population and enormous burden, disability is increasingly recognized as a human rights issue and a global public health issue.

Several international initiatives—such as the United Nations *Convention on the Rights of Persons with Disabilities* (CRPD) [5]—have been proposed to deal with this development priority. At the 67^th^ World Health Assembly in 2014, the WHO launched a global disability action plan, called the *better health for all people with disability*, to improve the health of this disadvantaged population in the world [6]. Undoubtedly, in order to achieve this arduous goal, it is fundamental to understand and analyze the health and health disparities of persons with disabilities.

Over the years, the number of studies on health disparities between people with and without disabilities has been increasing. These studies indicated that people with disabilities experience health disparities and greater unmet needs in comparison to the general population [7, 8]. For example, arthritis, asthma, cardiovascular disease, diabetes, obesity, oral disease, and stroke are highly prevalent among people with disabilities [9–11].

In fact, persons with disabilities have diverse and heterogeneous characteristics. They can be divided into many subgroups, based not only on demographic and socioeconomic characteristics, but also on disability conditions, such as disability type, severity, or reason. Therefore, health conditions may differ substantially within the population with disabilities.

However, there are significant gaps in research on health disparities within the population with disabilities [12]. Previous relevant studies focused on the US population and some particular health outcomes, and their definitions of disability as well as its types varied widely [13–16]. Inadequate information is available about health disparities among persons with disabilities in developing countries, such as China. Consequently, more research is needed to understand which subgroups of people with disabilities fare worse and are potentially more vulnerable than others, especially from developing countries.

In China, the second large-scale nationally representative household survey on disability was conducted in 2006. It reported that about 82 million people had one or more disabilities that affected their daily lives and social activities [17]. The weighted prevalence of disability increased over 20 years in China [18]. With an aging population, an increase in the prevalence of disabilities is expected [19]. In a study by Loyalka et al., it was found that the negative relationship between household income and disability in China was strong [20]. However, little is known about the health and health disparities of the disabled population in this developing country.

Therefore, the aim of our study was to make full use of existing data regarding the health of people with disabilities in a municipality of China to examine health disparities by type of disability. First, we evaluated the prevalence of some health conditions among adults with different types of disabilities. Then, the number of health conditions variables in each subgroup was determined. Finally, we examined the impact of controlling for demographic and severity covariates when analyzing the association between disability type and health outcome variables.

## Methods

### Ethics statement

The data in the current study were derived from the daily work of the Shanghai Disabled Persons’ Federation (SHDPF), the local organization for persons with disabilities. Prior consent was obtained from the SHDPF regarding the use of data from their information platform. Research ethics approval for this study was granted by the Institutional Review Board (IRB) of the School of Public Health, Fudan University (IRB#2015-08-0563). The requirement for informed consent was waived by the ethics board.

### Data source

The Shanghai Disabled Persons’ Rehabilitation Comprehensive Information Platform (SHDPRCIP), established by the SHDPF, was initiated to collect health and rehabilitation data of people with disabilities in Shanghai. Inclusion in the registry is voluntary. Entry in the registry, however, requires professional medical and functional evaluation based on the International Classification of Functioning, Disability, and Health (ICF) by qualified doctors. The type and severity of the disability are certified and classified after the evaluation. By the end of 2014, about 400,000 disabled persons were registered and managed by the SHDPF. This accounted for about half of the estimated number of people with disabilities in Shanghai.

Health examination, as a part of the comprehensive health and rehabilitation service package that is paid for by the SHDPF, was started in 2004 to improve the health of people with disabilities in Shanghai. Over the past few years, an increasing number of such people have been covered. In 2011, the SHDPF implemented initiatives to ensure a health examination for disabled persons every three years. At the beginning of every year, a comprehensive demand survey was conducted by the SHDPF in each district of Shanghai. Based on this database, registered disabled individuals who needed a health checkup were arranged, in batches according to their registration number, to attend a medical institution for examination every three years. Two province-level (Shanghai is a municipality directly under control of the central government) rehabilitation centers provided professional examination for about 40,000 disabled persons every year. The other 30,000 persons were sent to other recognized medical institutions in each district. So far, health examination data from the two centers have been transferred to the SHDPRCIP.

The health checkup for people with disabilities was composed of physical, imaging, and laboratory examinations. Physical examination included basic measurements (height, weight, blood pressure, etc.) and organ checkup (heart, lung, liver, spleen, anorectum, eye, ear, nose, throat, etc.). Imaging examination incorporated abdominal ultrasonic scan (liver, gallbladder, spleen, pancreas), electro cardiogram (ECG) and chest X-ray. Laboratory examination comprised routine blood examination, blood biochemical examination, routine urine examination, and immunological examination. During the checkup, basic results were first collected by a medical practitioner in each department. Then, a principal doctor synthesized all information, including laboratory results, to give the final report for each examinee.

Health checkup data in the SHDPRCIP from July 1, 2014 to June 30, 2015 were included in our study. For people who underwent 2 or more examinations because of serious diseases, we included only the latest data. We chose to include only working-age (18–64 years) adults with disabilities in our analysis because substantial health-related changes may exist in adults above 65 years of age. We excluded records with missing data on the variables of interest.

### Measures

#### Dependent variables

Based on the top diseases oriented health plan of the SHDPF[21], we chose five specific diseases of highest prevalence among this population from among the several kinds of abnormalities observed during the examinations. These diseases can be classified into the Tenth Revision of the International Classification of Diseases (ICD-10), including fatty liver (K76.0), ocular fundus arteriosclerosis (I70.8), chronic pharyngitis (J31.2), refractive error (H52.3), and hepatic cysts (K76.8). We focused on the health conditions of the entire population with disabilities; hence, those from gynecology examination were not included. Hemorrhoid was excluded because many people refused anorectal examination. All diseases were diagnosed by the doctors of the rehabilitation centers through disease history inquiry and physical or imaging examinations.

In addition, we selected five key risk factors for chronic conditions which were frequently observed, including abnormal ECG results, high Body Mass Index (BMI), high blood pressure, high blood glucose levels, and high blood lipid levels. Abnormal ECG results were considered a manifestation of cardiovascular disease and have been used in a previous population health research of disabled people in Shanghai [22]. The other four risk factors had significantly high burden in China [23]. Abnormal ECG findings were defined as evidence of ST-T change, T wave change, tachycardia, bradycardia, arrhythmia, myocardial ischemia, conduction blockade, high voltage, low voltage, etc. BMI ≥ 24 kg/m^2^ was considered high, which is viewed as overweight or obese according to the suggested guidelines in the Chinese population [24]. High blood pressure was defined as systolic blood pressure ≥ 140 mmHg and (or) diastolic pressure ≥ 90 mmHg. High blood glucose levels were defined as fasting blood glucose ≥ 6.1 mmol/L. High blood lipid levels were defined as total cholesterol or triglyceride levels higher than the particular center’s standard.

#### Primary independent variable

Under the stewardship of the Chinese Disabled Persons’ Federation, people with disabilities were divided into seven types based on the ICF, including visual, hearing, speech, physical, intellectual, mental, and multiple disability. Persons with multiple disabilities had two or more disability types. In our study, we combined the hearing impairment population with the speech disability population, as in the study by Zheng et al [18].

#### Covariates

Five basic demographic parameters were included as covariates in the adjusted model: gender (male or female), age (18–29, 30–39, 40–49, 50–59, and 60–64 years), hukou (a Chinese household registration system which has two types—rural and urban), education (elementary school or lower, middle school, high school, college, or higher), and marital status (never married, married, divorced, or widowed). Furthermore, individuals with severe disability might fare worse with regard to other health issues, so the severity of disability was also taken into account in this model. The disability level was classified into four levels by related function scores arrived at by using standard Chinese criteria based on the principles of the ICF: Level 1 indicating the most severe disability, Levels 2 and 3 indicating moderately severe to moderate disability, and Level 4 representing mild disability[25, 26]. The influence of these health conditions on individuals with disability has been rarely studied. To avoid over-adjustment, we did not use health outcome variables as covariates when modeling other health outcomes. We focused on demographic characteristics and disability severity as potential confounders. All covariates above were classified as categorical variables.

### Statistical analysis

All statistical analyses were performed with SPSS version 22. Basic descriptive statistics were used to present the demographic profile of the sample. The prevalence and number of diseases and risk factors were also compared between subgroups with different types of disabilities. A binary logistic regression model was used to examine the disparities in diseases and risk factors of interest. Logistic regression was used from two perspectives: 1) basic differences, unadjusted for other factors; and 2) differences after adjusting for key demographic covariates. Preliminary analyses showed the Variance Inflation Factor (VIF) values of the independent variables to be < 5, indicating that the multicollinearity issue did not exist [27]. Individuals with hearing and speech disability were considered the reference group to which groups with other types of disabilities were compared, because basic evaluation indicated that this group had more positive health outcomes. A p-value < 0.05 was considered statistically significant.

## Results

During the study period, 41,127 disabled persons visited rehabilitation centers for health examination; some of them underwent more than one checkup. In our analysis, we included the latest examination results from all 42,237 records in the SHDPRCIP. After excluding persons ages < 18 or ≥ 65 years (n = 9032) or with missing data on identified variables (n = 1013), we had an analytical sample of 31,082 persons with disabilities.

The demographic and disability characteristics of the total 31,082 persons with disabilities are presented in Table 1. The study population had an equal proportion of men (52.3%) and women (47.7%). The majority (47.4%) of the subjects were between 50 and 59 years of age, the average age being 53.1 ± 9.5 years. Most subjects were married (79.0%) and having an urban hukou (80.3%). Nearly half (51.9%) of the population were middle school-educated individuals, and 23.8% were high school-educated. In this sample, 49.3% of the subjects had physical disability, followed by visual limitation (21.7%) and intellectual disability (12.9%). Most of the individuals (79.2%) were classified as having a mild or moderate level of severity.

**Table 1:** Demographic and disability characteristics of sample.

Characteristics	n	%
Gender		
Male	16262	52.3
Female	14820	47.7
Age(year)		
18–29	1138	3.7
30–39	2329	7.5
40–49	4191	13.5
50–59	14731	47.4
60–64	8693	28.0
Marital Status		
Never married	4643	14.9
Married	24558	79.0
Divorced or widowed	1881	6.1
Education		
Elementary school or lower	6476	20.8
Middle school	16118	51.9
High school	7392	23.8
College or higher	1096	3.5
Hukou		
Rural	6137	19.7
Urban	24945	80.3
Disability type		
Hearing and speech	2917	9.4
Visual	6747	21.7
Physical	15336	49.3
Intellectual	4002	12.9
Mental	1722	5.5
Multiple	358	1.2
Disability severity		
Level 1	2384	7.7
Level 2	4109	13.2
Level 3	9435	30.4
Level 4	15154	48.8

Percentage may not total 100 due to rounding errors.

The age-standardized prevalence of health conditions in each disability group is displayed in Table 2. Health outcomes were not distributed evenly across the disability groups. Fatty liver was the most common disease (52.7%), especially in the mental disability group. A high proportion of people with physical or intellectual disability presented with ocular fundus arteriosclerosis. People with visual impairment had the highest rate of refractive error (60.0%). Among the risk factors, a BMI ≥ 24 was the most common health issue (48.9%), followed by high blood lipid levels (45.1%). Individuals with mental disability had the highest prevalence of BMI ≥ 24, high blood glucose levels, and high blood lipid levels, while those with hearing and speech impairment had the lowest prevalence. People with multiple disabilities had the highest prevalence of high blood pressure (38.3%).

**Table 2:** Health conditions among each disability type.

Health outcome	All disability, n (%)	Disability type, n (weighted %)					
		Hearing & speech	Visual	Physical	Intellectual	Mental	Multiple
Fatty liver	13704(44.1)	1171(40.0)	3009(43.8)	6893(43.8)	1581(39.5)	898(52.7)	152(45.5)
Ocular fundus arteriosclerosis	11449(36.8)	996(34.1)	1832(24.1)	7064(42.3)	946(43.4)	521(39.6)	90(30.1)
Chronic pharyngitis	8032(25.8)	738(25.5)	1873(27.9)	3900(25.8)	970(19.0)	439(25.3)	112(29.9)
Refractive error	7056(22.7)	347(12.0)	4181(60.0)	1682(11.4)	460(9.2)	267(14.6)	119(34.7)
Hepatic cysts	3570(11.5)	346(11.8)	932(12.4)	1914(11.5)	225(9.1)	127(9.1)	26(9.3)
Abnormal ECG	10423(33.5)	889(30.5)	2070(29.6)	5233(34.0)	1470(40.0)	648(38.2)	113(30.1)
BMI≥24	15191(48.9)	1278(43.8)	3074(45.4)	7646(48.9)	2006(50.2)	1023(57.7)	164(48.1)
High blood pressure	11244(36.2)	1020(34.8)	2663(37.0)	5818(35.9)	1154(38.2)	467(31.2)	122(38.3)
High blood glucose	5571(17.9)	473(16.1)	1342(18.6)	2709(16.6)	601(19.8)	389(25.3)	57(18.2)
High blood lipid	14018(45.1)	1242(42.4)	3022(43.8)	7259(45.9)	1503(43.7)	842(49.2)	150(45.1)

**Fig 1:**
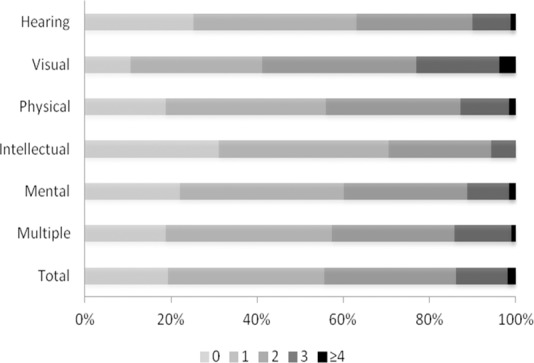
Number of diseases among people with different disability types.

**Fig 2:**
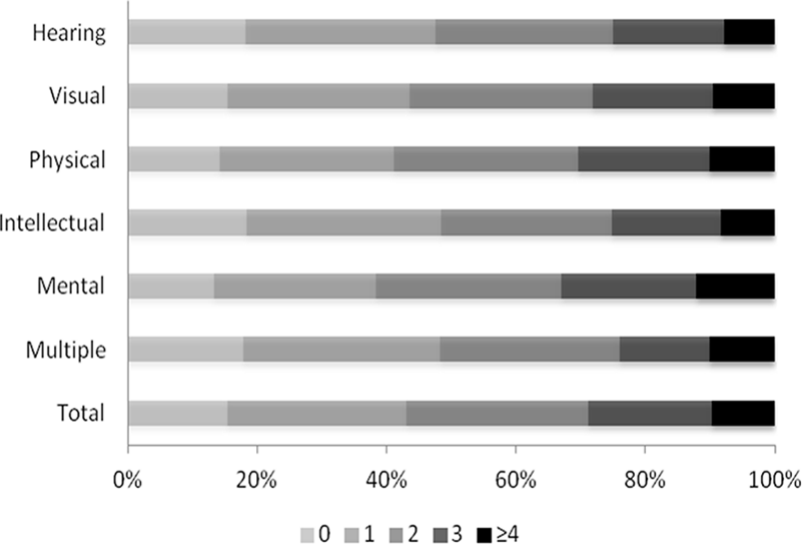
Number of risk factors among people with different disability types.

Table 3 shows that the mean number of diseases in the population was 1.41 ± 0.99. The mean number of diseases was the highest in the group with visual disability (1.75 ± 1.01), and lowest in the group with intellectual impairment (1.04 ± 0.89). The average number of risk factors per person was 1.82 ± 1.23 for the entire population. In group-wise classification, individuals with mental disability had the highest number of risk factors (1.96 ± 1.26), while those with hearing and speech limitations had the smallest number (1.68 ± 1.21). The number of diseases or risk factors we measured ranged from one to five, and varied across the different categories of disabilities (Figs 1 and 2). About 80% of the subjects had at least one disease of our interest. Among individuals with visual impairment, more than 20% had three or more diseases. Only 15% of the sample had none of the risk factors of interest, while more than 30% of the subjects with mental or physical disability had three or more risk factors.

**Table 3:** Mean number of health conditions among each disability type.

Health	All, n(SD)	Disability type, n(SD)					
outcome		Hearing*	Visual	Physical	Intellectual	Mental	Multiple
Diseases	1.41(0.99)	1.23(0.97)	1.75(1.01)	1.40(0.97)	1.04(0.89)	1.31(0.97)	1.39(0.97)
RF	1.82(1.23)	1.68(1.21)	1.80(1.23)	1.87(1.23)	1.68(1.23)	1.96(1.26)	1.69(1.24)

* Hearing and speech

RF, risk factors; SD, standard deviation

Table 4 shows the results of the regression analyses. In both models, people with visual disabilities were significantly less likely to have ocular fundus arteriosclerosis than those with hearing and speech impairment. However, in both the models, the visual impairment group was significantly more likely to have fatty liver, refractive error, high blood glucose levels, and high blood lipid levels than the hearing and speech impairment group. The visual impairment group was also more likely to have chronic pharyngitis, hepatic cysts, and high blood pressure in the unadjusted model; however, these were no longer statistically significant after adjustment for demographic factors.

**Table 4:** Health disparities by different disability type in unadjusted and adjusted model.

Health outcome	Disability type	Unadjusted model			Adjusted model		
		OR	95% CI	*p*	OR	95% CI	*p*
Fatty liver	Hearing & speech	Reference					
	Visual	1.20	1.10–1.31	<0.01	1.11	1.01–1.21	0.03
	Physical	1.22	1.12–1.32	<0.01	1.17	1.07–1.28	<0.01
	Intellectual	0.97	0.88–1.07	0.59	1.18	1.06–1.32	<0.01
	Mental	1.62	1.44–1.83	<0.01	1.75	1.54–1.98	<0.01
	Multiple	1.10	0.88–1.37	0.40	1.16	0.93–1.46	0.19
Ocular fundus arteriosclerosis	Hearing & speech	Reference					
	Visual	0.72	0.65–0.79	<0.01	0.60	0.55–0.67	<0.01
	Physical	1.65	1.52–1.79	<0.01	1.50	1.36–1.64	<0.01
	Intellectual	0.60	0.54–0.66	<0.01	1.33	1.17–1.51	<0.01
	Mental	0.84	0.74–0.95	0.01	1.41	1.21–1.63	<0.01
	Multiple	0.65	0.50–0.83	<0.01	0.83	0.63–1.09	0.18
Chronic pharyngitis	Hearing & speech	Reference					
	Visual	1.13	1.03–1.25	0.01	1.01	0.91–1.12	0.87
	Physical	1.01	0.92–1.10	0.88	0.97	0.88–1.07	0.59
	Intellectual	0.94	0.85–1.05	0.31	0.90	0.80–1.03	0.12
	Mental	1.01	0.88–1.16	0.88	0.93	0.81–1.08	0.34
	Multiple	1.34	1.06–1.71	0.02	1.19	0.93–1.52	0.16
Refractive error	Hearing & speech	Reference					
	Visual	12.07	10.68–13.64	<0.01	10.18	8.96–11.57	<0.01
	Physical	0.91	0.81–1.03	0.14	0.73	0.64–0.83	<0.01
	Intellectual	0.96	0.83–1.12	0.61	0.97	0.82–1.15	0.70
	Mental	1.36	1.14–1.61	<0.01	1.19	0.99–1.43	0.07
	Multiple	3.69	2.88–4.72	<0.01	3.72	2.88–4.82	<0.01
Hepatic cysts	Hearing & speech	Reference					
	Visual	1.19	1.04–1.36	0.01	1.03	0.90–1.18	0.69
	Physical	1.06	0.94–1.20	0.35	0.97	0.85–1.11	0.65
	Intellectual	0.44	0.37–0.53	<0.01	0.90	0.74–1.10	0.31
	Mental	0.59	0.48–0.73	<0.01	0.79	0.64–0.99	0.04
	Multiple	0.58	0.38–0.88	0.01	0.71	0.46–1.08	0.11
Abnormal ECG	Hearing & speech	Reference					
	Visual	1.01	0.92–1.11	0.84	1.05	0.95–1.15	0.37
	Physical	1.18	1.08–1.29	<0.01	1.19	1.09–1.30	<0.01
	Intellectual	1.32	1.20–1.47	<0.01	1.34	1.20–1.51	<0.01
	Mental	1.38	1.21–1.56	<0.01	1.43	1.26–1.63	<0.01
	Multiple	1.05	0.83–1.33	0.67	1.07	0.84–1.36	0.57
BMI≥24	Hearing & speech	Reference					
	Visual	1.07	0.98–1.17	0.11	1.07	0.98–1.17	0.15
	Physical	1.28	1.18–1.38	<0.01	1.26	1.15–1.37	<0.01
	Intellectual	1.29	1.17–1.42	<0.01	1.35	1.21–1.51	<0.01
	Mental	1.88	1.66–2.12	<0.01	2.08	1.83–2.36	<0.01
	Multiple	1.08	0.87–1.35	0.47	1.11	0.89–1.38	0.37
High blood pressure	Hearing & speech	Reference					
	Visual	1.21	1.11–1.33	<0.01	1.09	0.99–1.20	0.08
	Physical	1.14	1.05–1.23	<0.01	1.05	0.96–1.14	0.33
	Intellectual	0.75	0.68–0.83	<0.01	1.11	0.99–1.25	0.09
	Mental	0.69	0.61–0.79	<0.01	0.85	0.74–0.97	0.02
	Multiple	0.96	0.76–1.21	0.74	1.15	0.90–1.46	0.26
High blood glucose	Hearing & speech	Reference					
	Visual	1.28	1.14–1.44	<0.01	1.22	1.08–1.38	<0.01
	Physical	1.11	1.00–1.23	0.06	1.06	0.95–1.19	0.29
	Intellectual	0.91	0.80–1.04	0.17	1.26	1.09–1.47	<0.01
	Mental	1.51	1.30–1.75	<0.01	1.78	1.52–2.08	<0.01
	Multiple	0.98	0.73–1.32	0.89	1.14	0.84–1.54	0.41
High blood lipid	Hearing & speech	Reference					
	Visual	1.09	1.00–1.19	0.04	1.11	1.01–1.21	0.03
	Physical	1.21	1.12–1.31	<0.01	1.23	1.13–1.34	<0.01
	Intellectual	0.81	0.74–0.89	<0.01	0.98	0.87–1.09	0.66
	Mental	1.29	1.14–1.45	<0.01	1.40	1.23–1.59	<0.01
	Multiple	0.97	0.78–1.21	0.81	1.10	0.88–1.38	0.40

OR, odds ratio; CI, confidence interval

People with physical disabilities were significantly more likely to have fatty liver, ocular fundus arteriosclerosis, abnormal ECG findings, BMI ≥ 24, and high blood lipid levels in both models. The association with blood pressure was statistically significant only in the unadjusted model. The intellectual limitation group was less likely to have hepatic cysts, high blood pressure, and high blood lipid levels in the unadjusted model; however, it did not differ significantly from the reference group in adjusted analyses. Meanwhile, this group had a significantly higher likelihood of having fatty liver, ocular fundus arteriosclerosis, and high blood glucose levels after adjusting for the covariates.

People with mental disabilities fared significantly worse than those in the reference group on the 6 health outcomes (fatty liver, ocular fundus arteriosclerosis, abnormal ECG findings, BMI ≥ 24, high blood glucose levels, and high blood lipid levels). Nevertheless, the mental limitation group fared better on two health variables—hepatic cysts and blood pressure. After adjusting for demographic characteristics, people with multiple disabilities were significantly more likely to have refractive error than those in the hearing and speech impairment group.

## Discussion

Using a unique dataset containing relatively exhaustive information, the health disparities among people with disabilities in Shanghai were explored. There are still substantial information gaps on the health of people with disabilities. However, our study has enriched literature on health-related differences between people with different types of disabilities by providing data from a reasonably large sample in China.

Our results revealed disparities in the health outcomes we measured between subgroups of people with different types of disabilities. Even after adjustment for some covariates, the differences remained significant for most health conditions. This finding was quite different from that of Horner-Johnson et al., who showed that many of the differences between different types of disabilities disappeared after adjusting for certain factors[13]. The first reason for the different findings might be that the definitions of disability varied widely, even in one country[12]. Second, our study was based on the disability registry system; however, we found many adults with multiple disabilities who were not included in the system [28]. Due to more severe dysfunction, people with multiple disabilities might have less access to information or are less likely to be enrolled in the registry. Although this disability subgroup seemed to be enjoying good health in our study, it is not possible to draw definitive conclusions. Third, the Horner-Johnson study included a greater number of covariates; however, we also considered some key demographic characteristics. Meanwhile, our adjusted models took into account disability severity, which may be an important and influential factor [29–31]. Hence, the differences in our study were specifically related to the type of disability, not to differences in severity that could potentially covary with each disability type. Fourth, the inclusion of different dependent variables on health status may also be an important reason for the discrepancy between the results of the two studies. More specific diseases, rather than disease categories, were included in the current study to evaluate the health status and disparity between people with different kinds of disabilities more concretely. In addition to the above, the discrepancy might be attributable to significant differences of dietary habit, physical activity, health services access, disability-related social support, and health policy between the two countries.

Due to a lack of studies investigating health-related disparities between people with different types of disabilities, our findings could only be compared with a limited number of studies. People with mental disabilities were less likely than those with other types of disabilities to have high blood pressure; this result was consistent with that of a previous study investigating one district of Shanghai [22]. However, significant differences with respect to abnormal ECG findings and high blood glucose levels were not found in that study. The major limitation of previous studies has been their small sample size, which was inadequate to reflect the actual situation of health-related disparities with respect to type of disability. Because of severe air pollution in China [32], the prevalence of chronic pharyngitis may be high and the disparity between different disability groups small, even after adjustment for demographic factors.

Among the health outcomes we analyzed, people with hearing and speech impairment had relatively more positive results, which was consistent with the findings of Horner-Johnson et al. This might be attributed to the use of hearing aids, an assistive device that can improve hearing [33]. Of note, there is a huge discount on hearing aids in Shanghai. Although individuals with intellectual disabilities were more likely to have risk factors than the reference group, they fared better on most identified diseases and had the largest proportion of disease-free individuals.

The other groups experienced relatively poor health in comparison with the hearing and speech disability group. Each group fared worse on several health outcomes. It is possible that the impact of disability, in terms of healthy lifestyle and access to healthcare, among other factors, may vary between different types of disabilities [34]. As a result, various health-related differences exist between the different groups in the population with disabilities. The group with physical disability fared worse than the reference group in most health outcomes we examined. Moreover, from the perspective of number of health conditions, this group had the second highest average number of diseases or risk factors per person among all groups. The leading causes of physical disability in China, such as cerebrovascular disease and osteoarthritis [18], may cause relatively more severe health problems. The effect of visual impairment on refractive error was quite high (OR = 10.18, *p* < 0.01); this was indeed the strongest association observed in the adjusted model. It is much more likely that refractive error was the cause of visual disability, since people with visual disability had poor binocular vision that was not able to be corrected. With a lack of complete information about examinees, physicians may have categorized refractive error and visual disability as separate conditions.

A key finding of the current study was that individuals with mental disability were more likely than other subgroups to report poor health outcomes from a comprehensive view. This group experienced the highest prevalence of four health outcomes. Compared with the hearing and speech impairment group in the adjusted model, significant disparities were still apparent across the six health outcomes for this group. Furthermore, the mean number of risk factors was greatest in this population (1.96). This finding may reflect the obstacles for improving the health of the increasing population with mental disorders. Although the enormous direct burden of mental issues has been recognized over the years [35], the physical health status of people with mental disabilities also requires urgent attention. This is because mental disorders often lead to other health-related issues [36]; for example, there is evidence that depression predisposes people to diabetes [37].

This study has some limitations. First, it was a registry-based study and a considerable number of disabled persons chose to remain outside the registry, especially people with multiple disabilities. Second, data for some individuals in the registry were unavailable, including those who were bedridden or chose not to participate in the health examination. However, we believe that in spite of the above-mentioned limitations, to some extent, our study could represent the situation in Shanghai because of the relatively large sample size in real world practice. Third, we only focused on ten health outcomes based on current policy and circumstance in our study. The findings might be different because of the outcomes selected. Furthermore, health conditions were not included as covariates when modeling other health outcomes. Although over-adjustment could be avoided, some confounding effects might still exist. Finally, our analyses were cross-sectional and did not explore the cause and effect. While people with disabilities may be more likely to have some health conditions, it is also possible that health conditions lead to some disabilities. Long-term longitudinal studies that collect more data are needed to understand the relationships between disability and health status.

## Conclusion

The health-related disparities between working-age adults with different types of disabilities remained significant even after controlling for key demographic indicators, thus illustrating the challenges for improving the health of this section of the society. This argues for collecting more data and conducting more rigorous research to understand the complex health status of populations with different types of disabilities in China and throughout the world.
